# A sandwich-type cluster containing Ge@Pd_3_ planar fragment flanked by aromatic nonagermanide caps

**DOI:** 10.1038/s41467-020-19079-z

**Published:** 2020-10-20

**Authors:** Hong-Lei Xu, Nikolay V. Tkachenko, Zi-Chuan Wang, Wei-Xing Chen, Lei Qiao, Alvaro Muñoz-Castro, Alexander I. Boldyrev, Zhong-Ming Sun

**Affiliations:** 1 Tianjin Key Lab for Rare Earth Materials and Applications, State Key Laboratory of Elemento-Organic Chemistry, School of Materials Science and Engineering, Nankai University, 300350 Tianjin, China; 2 Department of Chemistry and Biochemistry, Utah State University, 0300 Old Main Hill, Logan, UT 84322-0300 USA; 3 Grupo de Química Inorgánicay Materiales Moleculares, Facultad de Ingenieria, Universidad Autonoma de Chile, El Llano Subercaseaux, 2801 Santiago, Chile

**Keywords:** Coordination chemistry, Chemical bonding

## Abstract

Sandwich-type clusters with the planar fragment containing a heterometallic sheet have remained elusive. In this work, we introduce the [K(2,2,2-crypt)]_4_{(Ge_9_)_2_[*η*^6^-Ge(PdPPh_3_)_3_]} complex that contains a heterometallic sandwich fragment. The title compound is structurally characterized by means of single-crystal X-ray diffraction, which reveals the presence of an unusual heteroatomic metal planar fragment Ge@Pd_3_. The planar fragment contains a rare formal zerovalent germanium core and a peculiar bonding mode of *sp*^2^-Ge@(PdPPh_3_)_3_ trigonal planar structure, whereas the nonagermanide fragments act as capping ligands. The chemical bonding pattern of the planar fragment consists of three 2c-2e Pd-Ge σ-bonds attaching Pd atoms to the core Ge atom, while the binding between the planar fragment and the aromatic Ge_9_ ligands is provided by six 2c-2e Pd-Ge σ-bonds and two delocalized 4c-2e σ-bonds. The synthesized cluster represents a rare example of a sandwich compound with the heteroatomic metal planar fragment and inorganic aromatic capping ligands.

## Abstract (web summary)

Sandwich-type clusters containing heterometallic interlayers have remained elusive. Here, the authors present the structure of a cluster composed of two aromatic nonagermanide caps flanking an heterometallic Ge@Pd_3_ planar fragment, where the Ge center is formally zerovalent.

## Introduction

**Fig. 1:**
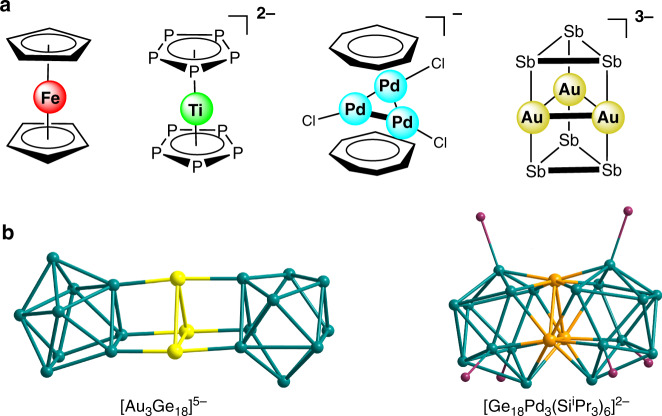
Schematic representations and structures of selected examples of known sandwich complexes. **a** Schematic representations of selected sandwich complexes. **b** Structures of analogous Ge-containing sandwich complexes. (Ge-atoms are green, Au-atoms are yellow, Pd-atoms are orange, and Si-atoms are purple).

Since the first sandwich complex (C_5_H_5_)_2_Fe, which was discovered in 1951, ferrocene and its derivatives have been the subject of intense research and many applications have been developed in chemical synthesis, catalysis, and materials science^1–4^. Inspired by this discovery, various organic cyclic π ligands were developed matching their orbitals symmetry with a metal center for generation of a vast array of metallocenes^5–8^. In 2002, an inorganic ligand *cyclo*-P_5_^−^ was applied for the complex [(P_5_)_2_Ti]^2−^ to stabilize a Ti(0) center (Fig. 1a)^9^. This compound represents the first all-inorganic sandwich complex and promoted the growth of an interdisciplinary research area. Besides the development of ligands, the types of interlayer have also been extended to the polyatomic metal core, and the representative examples are the [Pd_3_Tr_2_Cl_3_]^−^ (Tr = C_7_H_7_) and other analogous sandwich compounds containing different Pd interlayers^10–13^. Moreover, those complexes provide possibilities to broaden the applications of metallocenes in catalysis due to the catalytically active palladium. In addition, such metal monolayer compounds are suitable models for the construction of some bulky systems, for instance, metal-graphite-based materials^14^. An example of a sandwich complex that combines both a polyatomic interlayer and inorganic ligands is the all-metal cluster [Sb_3_Au_3_Sb_3_]^3−^, which furtherly broke prior limitation on the ligands and opened up more opportunities to build new types of sandwich compounds (Fig. 1a)^15^. Additionally, a sandwich-type cluster [Au_3_Ge_18_]^5−^ where a Au_3_ ring was flanked by two different Ge_9_ clusters further promoted the progress of ligands for sandwich compounds (Fig. 1b)^16^. The analogous structure was also presented in both 18-vertex *hypho*-deltahedron clusters [Ge_18_Pd_3_(E^i^Pr_3_)_6_]^2−^ (E = Si, Sn) with a Pd_3_-triangle inside, despite the broken Ge_9_ units (Fig. 1b)^17,18^. In this work, we report the synthesis and characterization of a sandwich-type anionic species {(Ge_9_)_2_[*η*^6^-Ge(PdPPh_3_)_3_]}^4−^ in which a trigonal planar fragment Ge@Pd_3_ is jammed between two aromatic Ge_9_ units. It is not only an extension of sandwich complex type to heteroatomic metal interlayer species, but also exhibiting an unusual stabilization mechanism of zerovalent main group elements.

## Results

### Synthesis and characterization

**Fig. 2:**
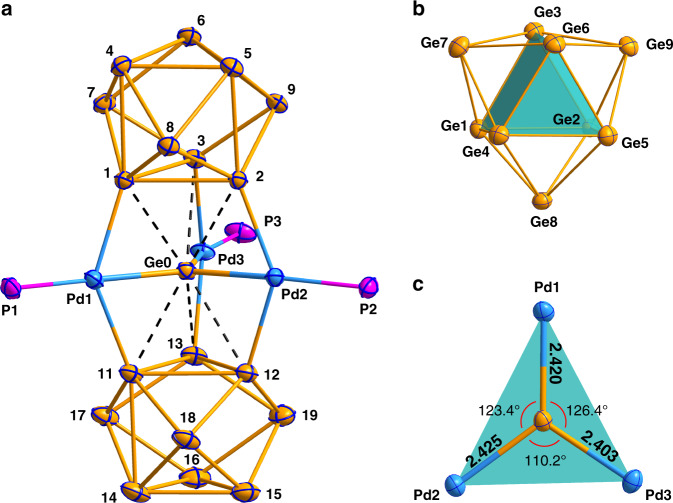
Structures of the cluster-1 {(Ge_9_)_2_[*η*^6^-Ge(PdPPh_3_)_3_]}^4-^ and its selected fragments. **a** The structure of the cluster-**1**, (H and C atoms are omitted for clarity, displacement ellipsoids with 35% probability). **b** The structure of Ge_9_ subunit. **c** The structure of planar Ge@Pd_3_ subunit. Interatomic distances are given in Å. Selected bond distances (angstroms) and angles (degrees): Ge1–Ge2: 2.744, Ge1–Ge3: 2.728, Ge2–Ge3: 2.718, Ge11–Ge12: 2.764, Ge12–Ge13: 2.751, Ge11–Ge13: 2.751, Ge4–Ge5: 2.670, Ge4–Ge6: 2.593, Ge5–Ge6: 2.619, Ge14–Ge15: 2.625, Ge14–Ge16: 2.612, Ge15–Ge16: 2.620, Ge2–Ge5: 2.808, Ge1–Ge4: 2.830, Ge3–Ge6: 3.061, Ge11–Ge14: 2.866, Ge12–Ge15: 2.893, Ge13–Ge16: 2.930 Pd1–Ge1: 2.475, Pd1–Ge11: 2.505, Pd3–Ge3: 2.533, Pd3–Ge13: 2.514, Pd2–Ge2: 2.482, Pd2–Ge12: 2.492. Ge1–Pd1–Ge11: 138.92, Ge1–Pd1–Ge0: 69.65, Ge11–Pd1–Ge0: 69.27, Ge2–Pd2–Ge12: 140.93, Ge2–Pd2–Ge0: 70.35, Ge12–Pd2–Ge0: 71.08, Ge3–Pd3–Ge13: 142.31, Ge3–Pd3–Ge0: 72.08, Ge13–Pd3–Ge0: 70.36.

The compound [K(2,2,2-crypt)]_4_{(Ge_9_)_2_[*η*^6^-Ge(PdPPh_3_)_3_]} was obtained by the reaction of an ethylenediamine (en) solution of K_4_Ge_9_ with (Triphenylphosphoranylidene)acetonitrile (NC – CPPh_3_) and Pd(PPh_3_)_4_ in the presence of 2,2,2-crypt (4,7,13,16,21,24-hexaoxa-1,10-diazabicyclo [8.8.8] hexacosane). These phosphines, such as PPh_3_, not only can act as useful ligands for organometallic compounds, but also are potential to be used as a mild oxidizing agent in Ge_9_ cluster chemistry based on the study of Sevov^19,20^. Here, the NC – CPPh_3_ was used, similar to PPh_3_, to change the reactivity of parent Ge_9_ cluster by decrease the negative oxidation state. The black needle-like crystals were observed in the test tube after two weeks (17% yield based on K_4_Ge_9_). The X-ray diffraction analysis (XRD) reveals that the complex crystallizes in the monoclinic space group *P*2_1_/*n* and the asymmetric unit contains two crystallographically distinct {(Ge_9_)_2_[*η*^6^-Ge(PdPPh_3_)_3_]}^4−^ anions with eight [K(2,2,2-crypt)]^+^ cations (Fig. 2 and Supplementary Fig. 2). The anion {(Ge_9_)_2_[*η*^6^-Ge(PdPPh_3_)_3_]}^4−^ exhibited a specific sandwich structure in which heterometallic Ge@(PdPPh_3_)_3_ planar fragment located between the two Ge_9_ subunits with a nonlinear *μ*_3_-*η*^1^:*η*^1^:*η*^1^-coordination mode (Fig. 2a).

Considering very similar structural characteristics for the two individual anionic clusters {(Ge_9_)_2_[*η*^6^-Ge(PdPPh_3_)_3_]}^4−^ (1 and 2, see in the Supplementary Fig. 2 and Supplementary Data 1), the following discussion will mainly focus on cluster-**1** and the significant differences will be appropriately pointed out. In the title cluster-**1**/**2**, two Ge_9_ subunits (A: Ge1–9 for **1**, Ge21–29 for **2**; B: Ge11–19 for **1**, Ge31–39 for **2**) possess almost identical shapes, which can be described as a *quasi*-*D*_3h_ symmetric tricapped trigonal prism (Fig. 2b). In the subunit B, the opposite triangular surfaces of the prism are nearly parallel with a very small dihedral angle of 1.38 (1.26 for **2**, degrees), while the A has more obvious deviation due to a larger angle value of 5.98 (5.44 for **2**, degrees). Analysis of the structural distortions of each Ge_9_ subunit in comparison to an ideal *D*_3h_-tricapped trigonal prism (ttp) and *C*_4v_-capped square antiprism (csa) was made by using the CShM code^21,22^. These results show a deviation of 0.549 Å (root-mean-square, rms) and 0.480 Å rms, for each subunit, in relation to a *D*_3h_-ttp structure, and a larger deviation in comparison to *C*_4v_-csa (1.218 and 1.522 Å rms). Thus, each subunit retains a *closo-D*_3h_ character, showing small distortions in comparison to an ideal ttp deltahedron.

In the Ge_9_ subunits of cluster-**1**, the lengths of the prisms (A: Ge1–Ge4, 2–5, 3–6: 2.808–3.060 Å, the longest one 3–6; B: Ge11–Ge14, 12–15, 13–16: 2.866–2.930 Å, the longest one 13–16) are elongated compared with those (2.71–2.73 Å) in bare [Ge_9_]^2–^ cluster with tricapped trigonal prismatic structure^23^, and which are in good agreement with the corresponding values of similar Ge_9_ subunits in cluster-**2** (A: Ge21–Ge24, 22–25, 23–26: 2.817–3.042 Å, the longest one 22–25; B: Ge31–Ge34, 32–35, 33–36: 2.860–2.913 Å, the longest one 32–35)). Such elongation of prism lengths also exists in the reported [Au_3_Ge_18_]^5–^ (2.879–3.027 Å)^16^. This kind of prism lengths extension may be closely related to the interaction between the Ge_9_ subunits and the Ge@Pd_3_ moiety. In order to better describe the shapes of nine-atom Ge clusters, a related ratio *h*/*e* where h—mean prism height and e—mean edge length is calculated for the distorted trigonal prism of A (Ge1, Ge2, Ge3, Ge4, Ge5, Ge6) and B (Ge11, Ge12, Ge13, Ge14, Ge15, Ge16), which shows the distortions away from an idealized limit^16,24^. The values of *h*/*e* in A and B are almost identical, ~1.08, fitting in with that (1.08 for two corresponding Ge_9_ subunits) in cluster-**2**, which is very close to—[Ge_9_^2−^]—(1.07)^25^ and [Ge=Ge_9_=Ge_9_]^6−^ (~1.10)^19^, and lies in the range of those in [Ge_9_]^2−^ (~1.00)^23^, [Ge_9_–Ge_9_]^6−^ (1.12)^26^ and [Au_3_Ge_18_]^5−^ (1.14)^16^. In addition, the six capping atoms (Ge7–9, Ge17–19) are not symmetrically located above the rectangular sides of the trigonal prism but are shifted in the direction of the planar fragment of binary Ge@Pd_3_ center. Such trend was also observed in the cluster-2, similar to the reported cluster [Au_3_Ge_18_]^5–16^. The distances (2.526–2.561 Å) between these capping Ge-atoms (Ge7–9 and Ge17–19) and the Ge-atoms adjacent to the Pd-atoms (Ge1–3 and Ge11–13) are significantly shorter compared with those of other Ge–Ge bonds in the cap (2.615–2.660 Å) in the cluster-**1**. Whereas the Ge–Ge distances (2.718–2.764 Å) within the two coordinated triangular faces (Ge1–3 and Ge11–13) are almost consistent with the corresponding triangular faces in the bare [Ge_9_]^2-^ cluster (2.73–2.759 Å)^23^, the lengths in other triangular faces (Ge4–6 and Ge14–16) are remarkably shorter (2.593–2.670 Å). Not surprisingly, the similar situation also occurs in the cluster-**2**.

In the peculiar planar fragment Ge@Pd_3_, the Pd–Ge bond lengths (av. 2.416 Å for **1**; 2.422 Å for **2**) are considerably shorter than those in other Pd–Ge bonds (av. 2.517 Å for **1**; 2.498 Å for **2**) between the Ge@Pd_3_ triangle and Ge_9_ fragments and even in other Pd–Ge cluster anions, such as [Ge_9_PdPPh_3_]^3−^ (2.54–2.64 Å)^27^, [Ni@(Ge_9_PdPPh_3_)]^2−^ (2.49–2.51 Å)^27^, [Pd_2_@Ge_18_]^4−^ (2.61–2.63 Å)^28^ [(Me_3_Si)Si]_3_EtGe_9_Pd(PPh_3_) (2.43–2.73 Å)^29^ and [Ge_18_Pd_3_(E^i^Pr_3_)_6_]^2−^ (E = Si, Sn) (Si: 2.457–2.741 Å; Sn: 2.447–2.728 Å)^17,18^. In turn, the Pd–Ge bond lengths in cluster-**2** lie in a narrower range (2.417–2.426 Å for Ge@Pd_3_; 2.486–2.525 Å for others) than corresponding those (2.403–2.425 Å; 2.475–2.533 Å) in cluster-**1**. The aforementioned values indicate that the Pd–Ge bonds are relatively strong in the Ge@Pd_3_ sheet of cluster-**1**/**2**. Additionally, the Pd–P distances (2.306–2.320 Å for **1**; 2.295–2.317 Å for **2**) are well comparable to that (2.306 Å) in [(Me_3_Si)Si]_3_EtGe_9_Pd(PPh_3_)^29^, but relatively longer than those in [Ge_9_PdPPh_3_]^3−^ (2.237 Å) and [Ni@(Ge_9_PdPPh_3_)]^2–^ (2.235 Å)^27^. The interplanar spacing between triangular faces (Ge1–3 and Ge11–13; Ge21–23 and Ge31–33) and Ge@Pd_3_ planar fragment are almost identical (**1**: 2.366 and 2.342 Å, respectively; **2**: 2.347 and 2.349 Å, respectively) and considerably short. In turn, in cluster-**1**/**2**, the bonding interaction between the two Ge_9_ moieties and the central Ge is very weak due to the long bond distances (**1**: 2.795–2.906 Å; **2**: 2.778–2.905 Å, see the Supplementary Data 1). The Pd···Pd distances, 3.959–4.325 Å for **1** and 3.988–4.260 Å for **2**, are far from the range of normal Pd−Pd bond lengths (2.53–2.70 Å)^30,31^, which means that there is no bond interaction between Pd-atoms.

**Fig. 3:**
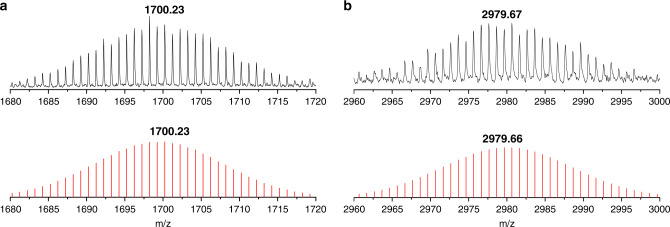
Negative-ion ESI mass spectra. **a** Spectrum of {(Ge_9_)_2_[*η*^6^-GePd_3_]}^−^ fragment. **b** Spectrum of {[K_3_(2,2,2-crypt)]{(Ge_9_)_2_[*η*^6^-Ge(PdPPh_3_)_3_]}^−^ fragment. Top: measured spectrum. Bottom: calculated spectrum.

In addition, the electrospray ionization mass spectrometry (ESI-MS) on a DMF solution of the crystals of [K(2,2,2-crypt)]_4_{(Ge_9_)_2_[*η*^6^-Ge(PdPPh_3_)_3_]} was performed in negative-ion mode to observe a series of fragments from the parent compound. Although several strong peaks were shown clearly and identified as {(Ge_9_)_2_[*η*^6^-GePd_3_]}^−^ (1700.23, Fig. 3a), {[K_3_(2,2,2-crypt)] {(Ge_9_)_2_[*η*^6^-Ge(PdPPh_3_)_3_]}^−^ (2979.67, Fig. 3b) and other parent compound peaks (Supplementary Fig. 4), any other peaks of small fragments were almost invisible in the range of the measured spectrum. The result may indicate the stability of title compound in solution.

### Structure and chemical bonding

The computationally optimized structure of {(Ge_9_)_2_[*η*^6^-Ge(PdPPh_3_)_3_]}^4−^ is in agreement with the X-Ray characterized structure denoting *η*^6^-Ge–Ge distances of 2.905 Å, *η*^6^-Ge–Pd of 2.462 Å, and Ge–Pd of 2.530 Å. The calculated Pd–Pd distances are 4.324 Å, which is larger than the sum of their van der Waals radius (~4.1 Å)^32^ indicating the absence of Pd–Pd two-center bonds. Thus, the central moiety can be better described as a *sp*^2^-Ge@(PdPPh_3_)_3_ trigonal planar structure, rather than a Pd_3_ ring. The electronic structure of {(Ge_9_)_2_[*η*^6^-Ge(PdPPh_3_)_3_]}^4−^ shows a sizable frontier orbital gap as a result of bringing together two *closo*-Ge_9_^2−^ units with a slightly distorted *D*_3h_ geometry mediated by a bridging moiety. Such behavior suggests that each *closo*-[Ge_9_]^2−^ unit in {(Ge_9_)_2_[*η*^6^-Ge(PdPPh_3_)_3_]}^4−^ exhibits spherical aromatic properties. Notably, it was previously shown, that the bare [Ge_9_]^2−^ shows spherically aromatic properties, and [Ge_9_]^4−^ clusters are locally σ-aromatic^33,34^.

**Fig. 4:**
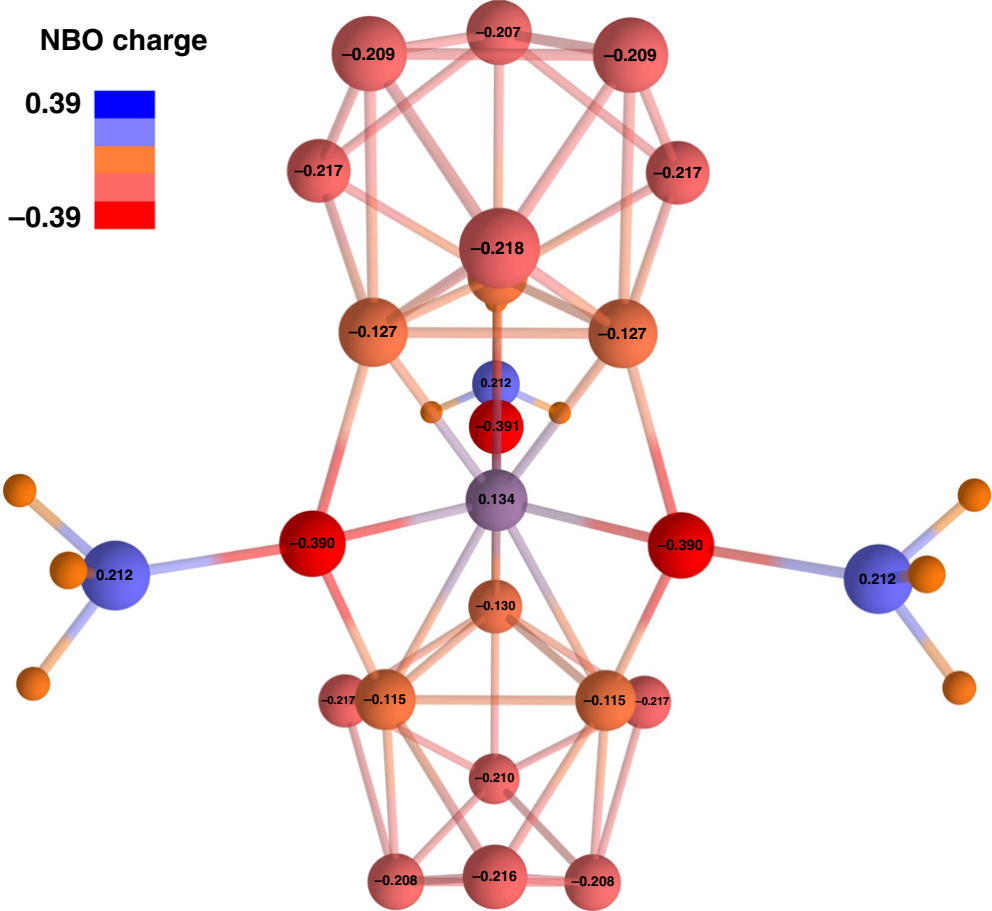
The charge distribution calculated via NBO method. Positive and negative charges are illustrated in a scale form blue to red, respectively. Lines between atoms help in visualization and do not represent 2c–2e bonds here and elsewhere. Charges of hydrogen atoms are omitted for clarity.

The presence of two [Ge_9_]^2−^ ligands and the overall charge of the complex −4 push us to the conclusion that the formal charge of the central Ge@(PdPPh_3_)_3_ fragment as 0. To show this, the charge distribution was calculated using the natural bond orbitals (NBO) analysis. The results are shown in Fig. 4. It was found that the overall charges of Ge_9_ fragments are −1.6 a.u., while the central Ge-atom bears slightly positive close to zero charge, which were classically found in organogermanium complexes and stabilized by carbine ligands that donate electron pairs into their empty orbitals^35–39^. Thus, the natural population analysis is in a good agreement with our assumption of zerovalent central Ge-atom stabilized by two [Ge_9_]^2−^ ligands. Notably, the Pd-atoms have a partially negative charge, which could be explained by the donor-acceptor nature of two-center two-electron (2c–2e) Pd–P bonds. In turn, the formal oxidation state of Pd-atoms is 0. The complete table with natural charges could be found in the Supplementary Information file (Supplementary Table 2).

**Fig. 5:**
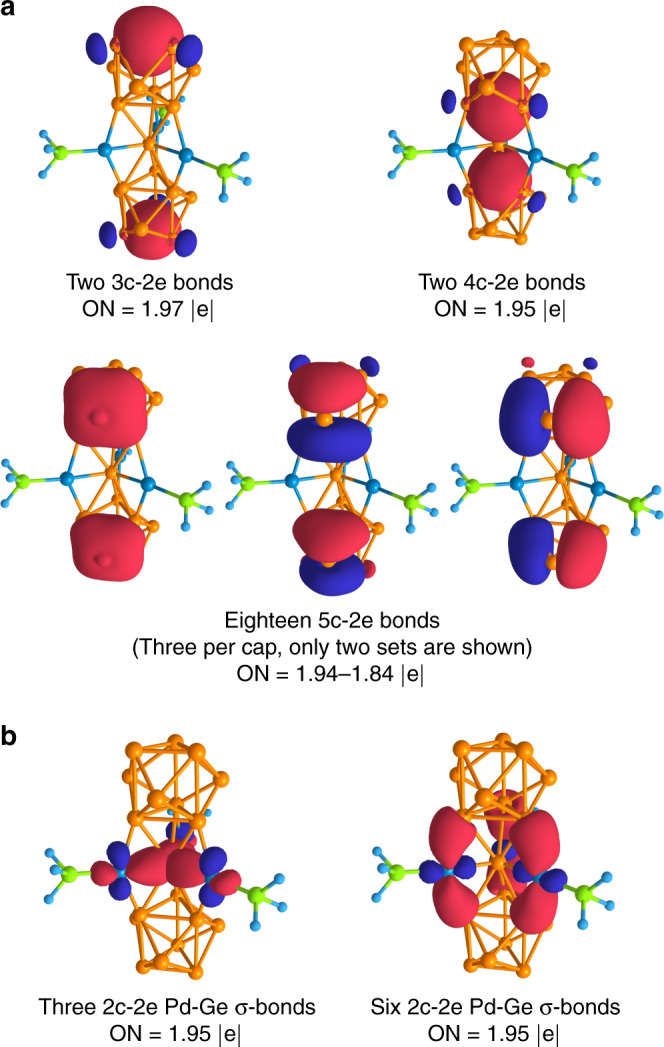
The result of AdNDP analysis of the selected fragments of the cluster-1. **a** Multicenter bonds of {(Ge_9_)_2_[*η*^6^-Ge(PdPH_3_)_3_]}^4−^. **b** Localized 2c–2e bonds of {(Ge_9_)_2_[*η*^6^-Ge(PdPH_3_)_3_]}^4−^.

To gain insights on the chemical bonding pattern of {(Ge_9_)_2_[*η*^6^-Ge(PdPPh_3_)_3_]}^4−^ cluster, we performed the adaptive natural density partitioning (AdNDP) analysis^40^ as implemented in the AdNDP 2.0 code^41^ of a model {(Ge_9_)_2_[*η*^6^-Ge(PdPH_3_)_3_]}^4−^ cluster (the phenyl substituents were replaced by hydrogen atoms; such substitution does not affect the bonding of the central fragment). The model complex contains 134 valence electrons, which can be localized to 67 two-electron bonding elements. Starting our search from one-center two-electron elements, the algorithm revealed the presence of lone pairs on the Germanium and Palladium atoms (Supplementary Fig. 15). Thus, twelve *s*-type lone pairs on Ge-atoms with occupancy numbers (ONs) 1.89–1.87 | e| and twelve *d*-type lone pairs (four lone pairs per each atom) on Pd-atoms with ONs 1.98–1.91 | e| were localized. Further localization showed the presence of twenty-one 2c–2e bonds (Fig. 5b, Supplementary Fig. 15). Predictably, we found a completely classical bonding pattern for PH_3_ groups with three 2c–2e P–H σ-bonds per each group. In turn, the PH_3_ groups attached to the Pd-atoms by 2c–2e bonds with ON = 1.99 | e | (contribution of P-atoms is ~86%). The planar fragment consists of three 2c–2e Pd–Ge σ-bonds attaching Pd-atoms to the central Ge-atom, and six 2c–2e Pd–Ge σ-bonds bind the Ge@Pd_3_ planar fragment and Ge_9_ fragments (contribution of Ge-atoms is ~77%). The binding interactions within the planar fragment found by the AdNDP are consistent with the ELF topology analysis (Supplementary Fig. 16).

Further molecular orbital analysis, as given by the molecular orbital diagram of the (Ge_9_)_2_^4−^-Ge(PdPPh_3_)_3_ interaction (Supplementary Figs. 19, 20), shows several bonding contributions involving mainly π-radial orbitals from the (Ge_9_)_2_^4−^ fragment. The HOMO orbital is given by the bonding interaction between a *p*_z_-Ge based orbital from the central Ge-atom, and the pertinent π-radial orbitals from the Ge_9_ clusters. A bonding interaction between Ge_9_ clusters and *d*-Pd-atoms could be seen from HOMO-2 and HOMO-3, which in turn enhance the σ-Ge@Pd_3_ interaction. Such bonding interactions are well summarized by the localized bonds provided by the AdNDP analysis, showing three σ-Ge@Pd bonds based on the *d*-Pd interacting orbitals with the Ge_9_ clusters, and six 2c–2e Pd–Ge (Fig. 5b).

Further localization showed that the Ge_9_ fragments possess σ-aromatic character (with locally σ-aromatic Ge_5_-caps and Ge_3_-triangles, Fig. 5a). Notably, almost identical chemical bonding pattern was described in our previous work for nonagermanide clusters^34^. The main difference is the presence of 4c–2e bonds that bound the central Ge-atom and two Ge_9_ units, which partially present in the HOMO and HOMO-1 (Supplementary Fig. 17). We want to note that the same delocalization with the formation of the 4c–2e bond (that contributes to bonding interaction) was found for copper-containing nonagermanide clusters such as Cu[Ge_9_{P(NH_2_)_2_}_3_], Cu(NHC)[Ge_9_{P(NH_2_)_2_}_3_]^−^, and {[Ge_9_{P(NH_2_)_2_}_3_]Cu[Ge_9_{P(NH_2_)_2_}_3_]}^−^^34^. Hence, the two 4c–2e bonds have the main role in the stabilization of the zerovalent germanium atom. As a result, a charge donation to the empty Ge-*p*_*z*_ atomic orbital occurs, favoring the sandwich-like incorporation and charge state stabilization.

**Fig. 6:**
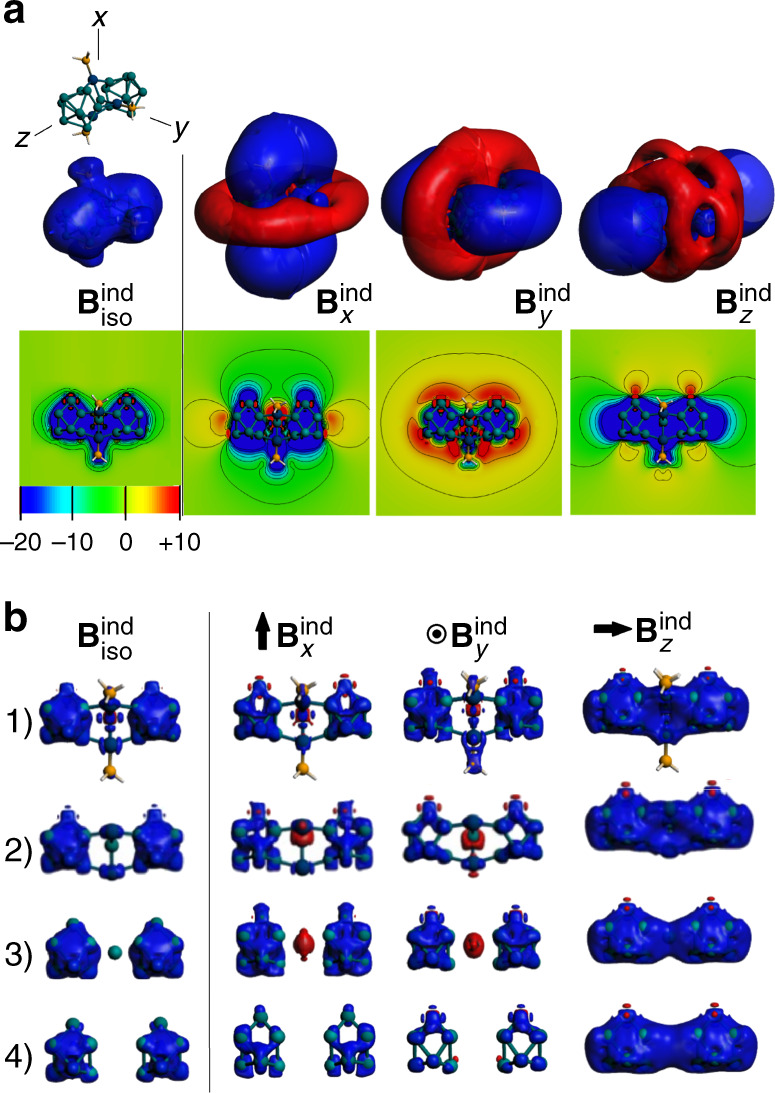
Plots and isosurfaces of magnetic response of the studied cluster. **a** Induced magnetic field of the studied cluster involving an orientation averaged (B^ind^_iso_) and orientations of the external field along three representative axes. Isovalue set to ± 2 ppm (top). The cut-plane representations are given (bottom). Blue: shielding; Red: Deshielding surfaces. **b** Shielding surfaces (35 ppm) for {(Ge_9_)_2_[*η*^6^-Ge(PdPH_3_)_3_]}^4−^ (1), the unligated core {(Ge_9_)_2_[*η*^6^-GePd_3_]}^4−^ (2), the core without Pd-atoms {(Ge_9_)_2_[*η*^6^-Ge]}^4−^ (3), and the non-bridged species [Ge_9_ Ge_9_]^4−^ (4).

Moreover, analysis of the magnetic response of {(Ge_9_)_2_[*η*^6^-Ge(PdPH_3_)_3_]}^4−^ reveals a spherical-like shielding surface at both Ge_9_ units as a characteristic feature of spherically aromatic compounds, as obtained from an orientation averaged applied field which accounts for the isotropic response (B^ind^_iso_) owing to the constant molecular tumbling in solution (Fig. 6a). The spherical aromatic characteristic of each Ge_9_ subunit is provided by the arrangement of locally σ-aromatic regions revealed by the AdNDP analysis (*vide supra*). The aromatic nature of nonegermanide subunits remains after the Ge_9_-Ge@Pd_3_ interaction, and their structural features are close to a *closo*-*D*_3h_-ttp cage. This result denotes that the overall cluster can be considered as two-spherical aromatic clusters held together by the central Ge@(PdPPh_3_)_3_. Under a specific orientation of the applied field (B^ind^_*x*_, B^ind^_*y*_, or B^ind^_*z*_), the distinctive shielding cone property for aromatic species is obtained at each Ge_9_ unit. For a perpendicular orientation in relation to the Ge_9_–Ge–Ge_9_ axis (B^ind^_*x*_ and B^ind^_*y*_), a shielding region is obtained which sum together in a common region of about −3.75 ppm (Figs. 6a, 2), which separate into independent parallel cones at isosurfaces above ±4 ppm together, being complemented with a deshielding region (Fig. 6b). Interestingly, for a parallelly oriented field along Ge_9_–Ge–Ge_9_ axis (B^ind^_*z*_), two shielding cones are obtained, which overlap the shielding region at the central Ge@(PdPPh_3_)_3_, originated from each Ge_9_ sides, denoting two separated complementary deshielding regions at the Ge_9_ belt.

From Fig. 6a, it is shown that {(Ge_9_)_2_[*η*^6^-Ge(PdPH_3_)_3_]}^4−^ is able to sustain a shielding cone upon different orientations of the applied field centered at each Ge_9_ unit, which is a distinctive feature of spherical aromatic compounds in contrast to planar counterparts, where is sustained under a parallel orientation (for example benzene, Supplementary Fig. 18). At different orientations, the shielding region is originated at each Ge_9_ unit, as can be seen from larger isosurface values (>±35 ppm, Fig. 6b) denoting the isotropic term (B^ind^_iso_), and from perpendicular orientation in relation to the Ge_9_–Ge–Ge_9_ axis (B^ind^_*x*_ and B^ind^_*y*_). Moreover, for a field oriented along the Ge_9_–Ge–Ge_9_ axis (B^ind^_*z*_) besides the shielding region (>−35 ppm) at each nonagermanide units, a shielding region involving each capped Ge_3_ face from Ge_9_ and the bridging Ge@Pd_3_ group was observed, which suggests a potential planar aromatic behavior in the Ge_3_–Ge@Pd_3_–Ge_3_ fragment as a result of the Ge_9_–Ge(PdPPh_3_)_3_–Ge_9_ bonding interaction. However, further inspection for {(Ge_9_)_2_[*η*^6^-Ge(PdPH_3_)_3_]}^4−^ in comparison to the unligated core {(Ge_9_)_2_[*η*^6^-GePd_3_]}^4−^, the core by removing Pd-atoms {(Ge_9_)_2_[*η*^6^-Ge]}^4−^, and the non-bridged species [Ge_9…_Ge_9_]^4−^, shows that the shielding contribution is originated from the spherical aromatic Ge_9_^2−^ units mainly, supporting the description of {(Ge_9_)_2_[*η*^6^-Ge(PdPPh_3_)_3_]}^4−^ as a cluster with two-bridged spherical aromatic units.

## Discussion

In summary, we report a synthesis of {(Ge_9_)_2_[*η*^6^-Ge(PdPPh_3_)_3_]}^4−^ that represents the peculiar sandwich-type species containing a heterometallic Ge@Pd_3_ planar fragment in which the germanium core of zero oxidation state is stabilized in the sandwich framework. Unlike prior weak metal–metal interactions in the interlayer, such as the Au_3_ ring of [Sb_3_Au_3_Sb_3_]^3–15^, the heterometallic Ge@Pd_3_ is formed by strong Pd–Ge bonding interactions, which may play a vital role in its properties. The AdNDP and ELF analyses reveal the presence of three 2c–2e bonds attaching Pd-atoms to the central Ge-atom in the Ge@Pd_3_ triangle and six 2c–2e bonds between Pd-atoms and two Ge_9_ units. Two 4c–2e bonds between Ge_9_ units and central Ge-atom have the main role in the stabilization of the zerovalent germanium. The analysis of the magnetic response exhibits that the overall cluster can be considered as two spherically aromatic fragments held together by the central Ge@(PdPPh_3_)_3_ group.

The {(Ge_9_)_2_[*η*^6^-Ge(PdPPh_3_)_3_]}^4−^ complex expands the borders of possible sandwich compounds showing that the metal interlayer can be formed by different metal elements, including transition metals and main group metals. We believe that the heterometallic planar fragment can bring some fascinating properties to new sandwich species, which provides more opportunities for new applications of sandwich complexes.

## Methods

### Synthesis of [K(2,2,2-crypt)]_4_{(Ge_9_)_2_[η^6^-Ge(PdPPh_3_)_3_]}

All manipulations and reactions were performed under a dry nitrogen atmosphere in a glove box. Ethylenediamine (Aldrich, 99%) and toluene (Aldrich, 99.8%) were freshly distilled and stored under nitrogen prior to use. 2,2,2-crypt (4,7,13,16,21,24-Hexaoxa-1,10-diazabicyclo (8.8.8) hexacosane, purchased from Acros, 98%), (Triphenylphosphoranylidene)acetonitrile (aladdin, >98%) and Pd(PPh_3_)_4_ (Alfa-Aesar, 99%) was dried in vacuum for 12 h prior to use. The precursor K_4_Ge_9_ was synthesized by heating a stoichiometric mixture of the elements (K: 386 mg, Ge: 1.614 g; K: + 99 %, Ge: 99.999 %, all from Strem) at a rate of 50 °C per hour to 900 °C and keeping it for 3 days in sealed niobium containers closed in evacuated quartz ampules. The furnace was slowly cooled to room temperature at a rate of 50 °C per hour^42^. The K_4_Ge_9_ solid was obtained with a high yield (~90%, 1.8 g) and stored under a dry nitrogen atmosphere in a glove box. In a glass vial, 81 mg (0.1 mmol) of K_4_Ge_9_ and 100 mg (0.27 mmol) of 2,2,2-crypt were dissolved in ethylenediamine (1.5 mL). After stirring for 15 min, the brown solution was filtered onto 36.2 mg (0.12 mmol) of (Triphenylphosphoranylidene)acetonitrile. After 0.5 h at 55 °C, the brown solution was added slowly to a 0.5 mL toluene solution of 92.4 mg (0.08 mmol) of Pd(PPh_3_)_4_ and vigorously stirred for 1 h at room temperature, and then another 92.4 mg of Pd(PPh_3_)_4_ was added. After 3 h at room temperature, the reaction mixture was filtered through glass wool and layered with 3 mL toluene. After 17 days, black needle-like crystals of [K(2,2,2-crypt)]_4_{(Ge_9_)_2_[*η*^6^-Ge(PdPPh_3_)_3_]} was observed in the test tube (33 mg, 17% yield based on K_4_Ge_9_).

### Theoretical methods

Geometry optimization and frequency calculations were performed using Gaussian 16 software package^43^. Optimized geometries, total energies are reported at the PBE0/Def2-TZVP level of theory^44,45^. The DFT wave functions were found to be stable, so the DFT approximation is valid. To understand the chemical bonding of investigated species, we carried out electron localization analysis at the same level of theory using the AdNDP method as implemented in the AdNDP 2.0 code^40,41^. ELF calculations were performed via MultiWFN software^46,47^. In addition, the isosurface and cut-plane representation of the induced magnetic field (B^ind^) was obtained within the GIAO formalism at the relativistic ZORA-PBE0/TZ2P level of theory by using the ADF suite unraveling the long-range characteristics of the magnetic response^48,49^.

### Crystallographic methods

Suitable crystals from the [K(2,2,2-crypt)]_4_{(Ge_9_)_2_[*η*^6^-Ge(PdPPh_3_)_3_]} were selected for X-ray diffraction analyses. Crystallographic data were collected on a Rigaku XtalAB Pro MM007 DW diffractometer (Cu–Mo Kα radiation) at 100 K. The structure of the crystal was solved using direct methods and then refined using SHELXL-2014 and Olex2^50–52^, in which all the non-hydrogen atoms were refined anisotropically. All hydrogen atoms of organic groups were rationally placed by geometrical considerations. The K2 and K8 were refined anisotropically and show an abnormal thermal motion that could not be resolved by using restraints. The limitation of data quality leads to the low bond precision on C–C bonds, and large cell volume also makes it not easy to obtain better data. The uncoordinated solvent molecules could not be modeled properly, so the PLATON SQUEEZE procedure was used during the refinement to remove the solvent molecules^53^.

### Energy dispersive X-ray (EDX)

EDX Analysis was performed using a scanning electron microscope (Hitachi S-4800) equipped with a Bruker AXS XFlash detector 4010. Data acquisition was performed with an acceleration voltage of 20 kV and an accumulation time of 150 s.

## Supplementary information

Supplementary Information

Description of Additional Supplementary Files

Supplementary Data 1

## Data Availability

The data that support the findings of this study are available from the corresponding authors on a reasonable request. The X-ray crystallographic coordinates for structures reported in this study have been deposited at the Cambridge Crystallographic Data Centre (CCDC), under deposition number 1997656. These data can be obtained free of charge from The Cambridge Crystallographic Data Centre via www.ccdc.cam.ac.uk/data_request/cif.
