# Supplementary material for: A sandwich-type cluster containing Ge@Pd3 planar fragment flanked by aromatic nonagermanide caps
Source: Nat Commun. 2020 Oct 20;11:5286. doi: 10.1038/s41467-020-19079-z (PMC7575547; doi:10.1038/s41467-020-19079-z)
Supplement: Supplementary file 1 — Supplementary Information [file 41467_2020_19079_MOESM1_ESM.pdf]

Supplementary Information

A Sandwich-Type Cluster Containing Ge@Pd_3 Planar Fragment Flanked by Aromatic Nonagermanide Caps

Xu *et al.*

Supplementary Figures

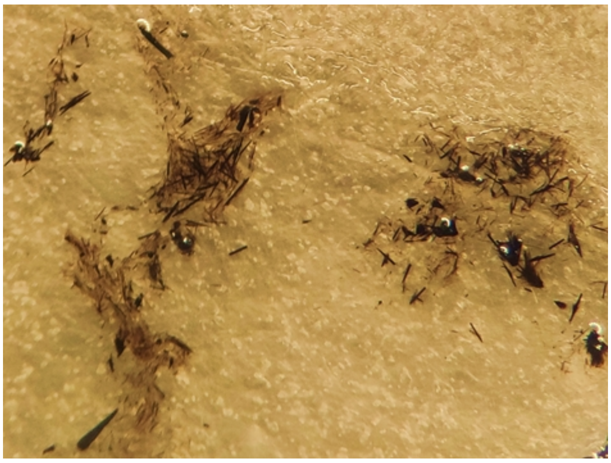

Supplementary Figure 1. Crystals of $[K(2,2,2\text{-crypt})]_4\{(Ge_9)_2[\eta^6\text{-}Ge(PdPPh_3)_3]\}$ dispersed in silicon oil.

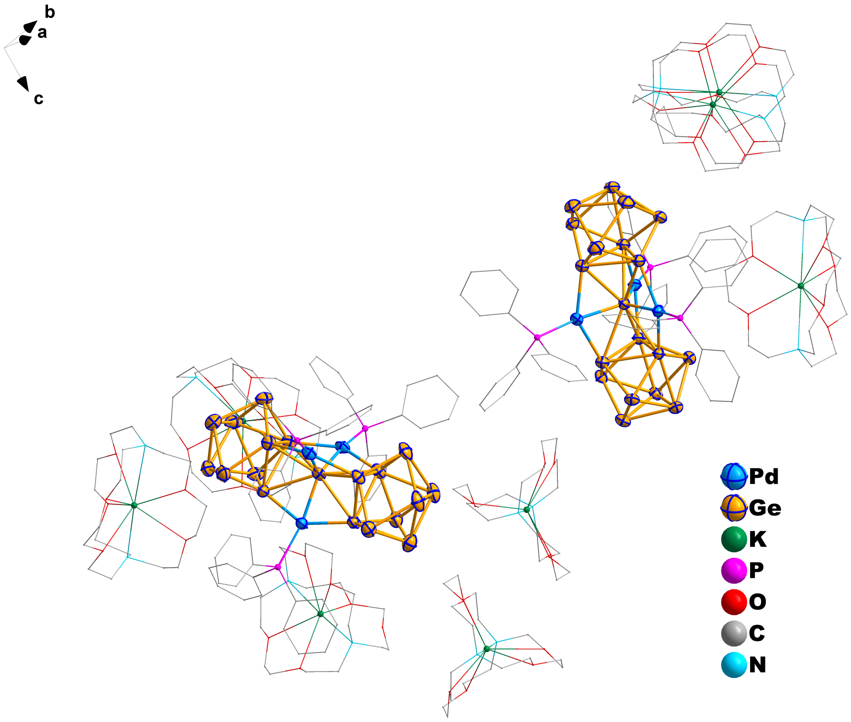

Supplementary Figure 2. Asymmetric unit of $[K(2,2,2\text{-crypt})]_4\{(Ge_9)_2[\eta^6\text{-}Ge(PdPPh_3)_3]\}$ with the cluster fragment. Thermal ellipsoids are drawn at 50% probability. The minor components are omitted for clarity.

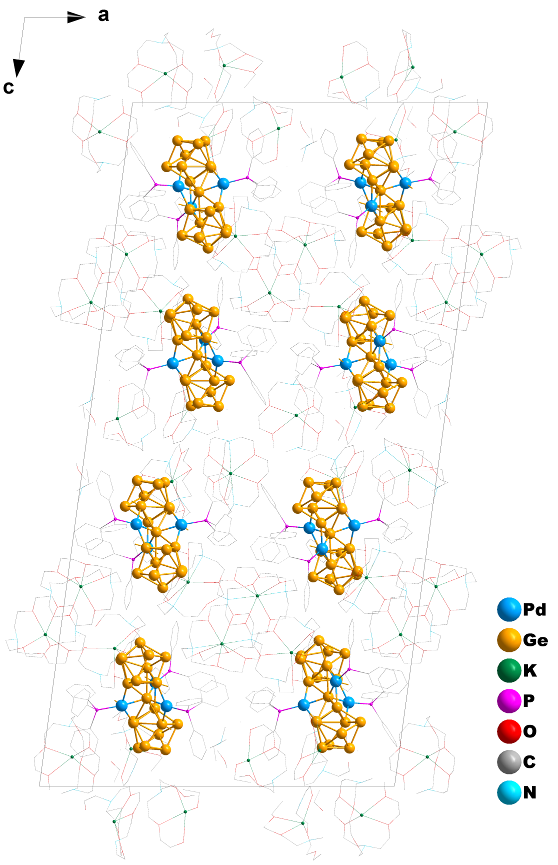

Supplementary Figure 3. Unit cell of $[K(2,2,2\text{-crypt})]_4\{(Ge_9)_2[\eta^6\text{-}Ge(PdPPh_3)_3]\}$. Minor component in the cluster site are omitted for clarity.

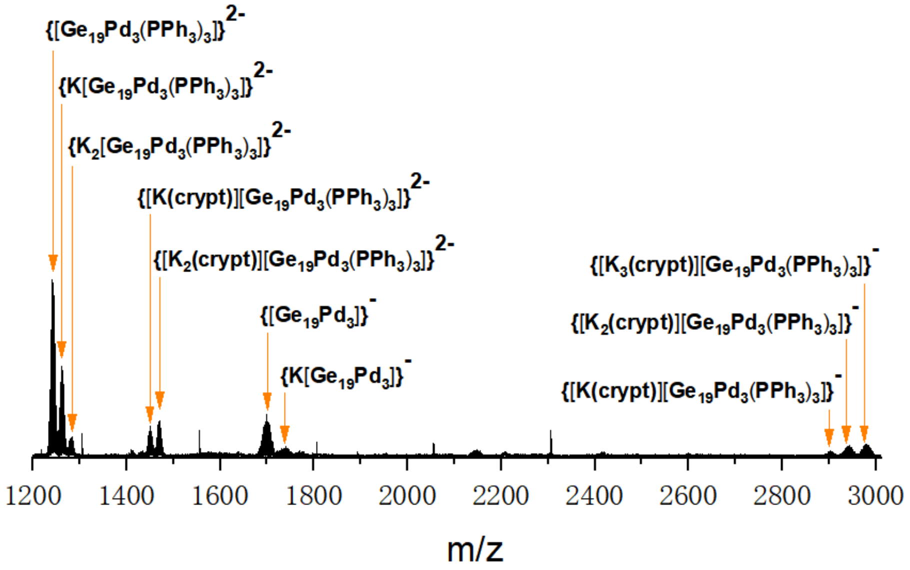

Supplementary Figure 4. Overview ESI mass spectrum in negative ion mode of a freshly dissolved crystalline sample of $[K(2,2,2\text{-crypt})]_4\{(Ge_9)_2[\eta^6\text{-}Ge(PdPPh_3)_3]\}$ in DMF.

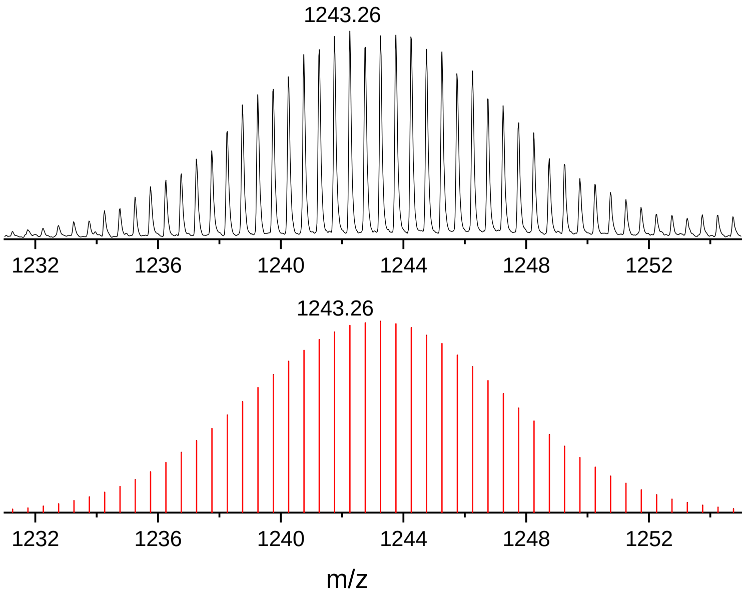

Supplementary Figure 5. Measured (top) and simulated (bottom) spectrum of the fragment $\{[Ge_{19}Pd_3(PPh_3)_3]\}^{2-}$.

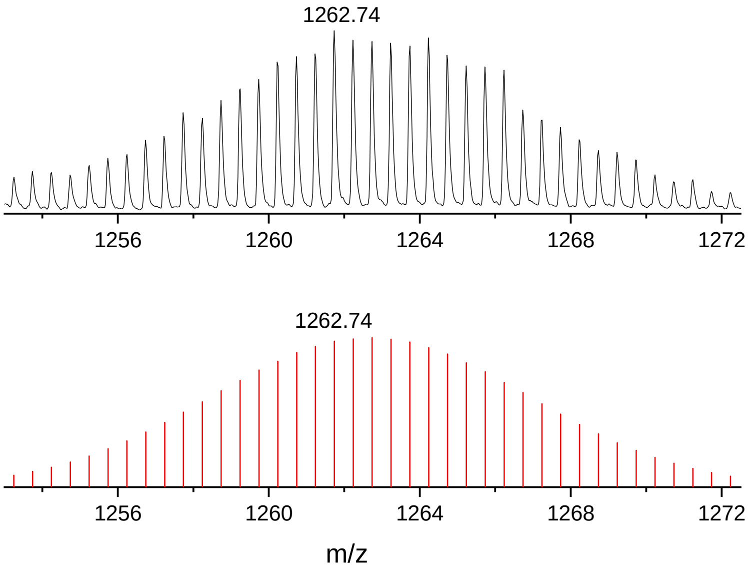

Supplementary Figure 6. Measured (top) and simulated (bottom) spectrum of the fragment $\{K[Ge_{19}Pd_3(PPh_3)_3]\}^{2-}$.

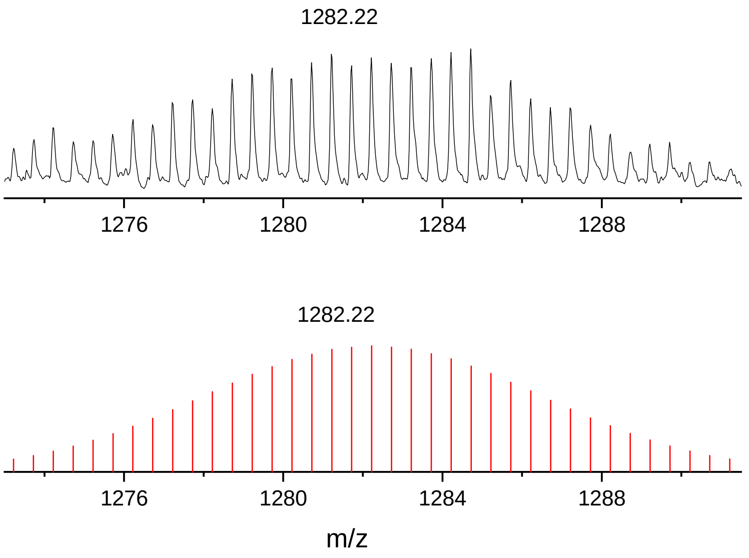

Supplementary Figure 7. Measured (top) and simulated (bottom) spectrum of the fragment $\{K_2[Ge_{19}Pd_3(PPh_3)_3]\}^{2-}$.

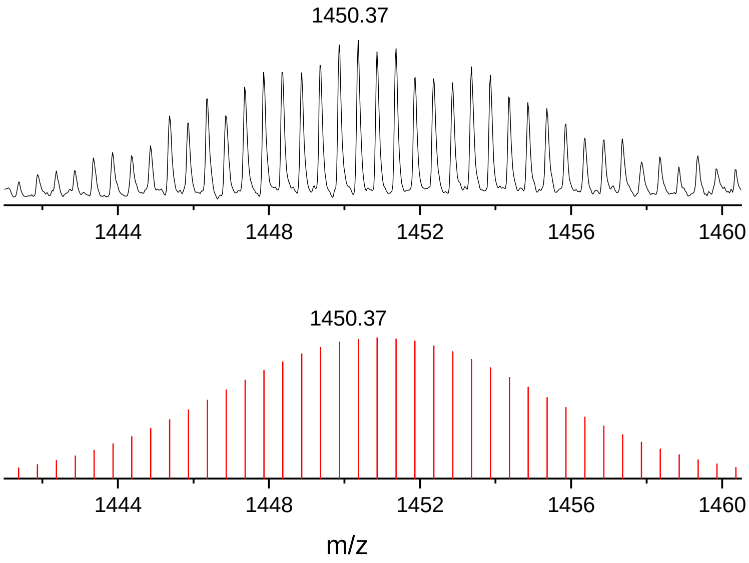

Supplementary Figure 8. Measured (top) and simulated (bottom) spectrum of the fragment $\{[K(2,2,2\text{-crypt})][Ge_{19}Pd_3(PPh_3)_3]\}^{2-}$.

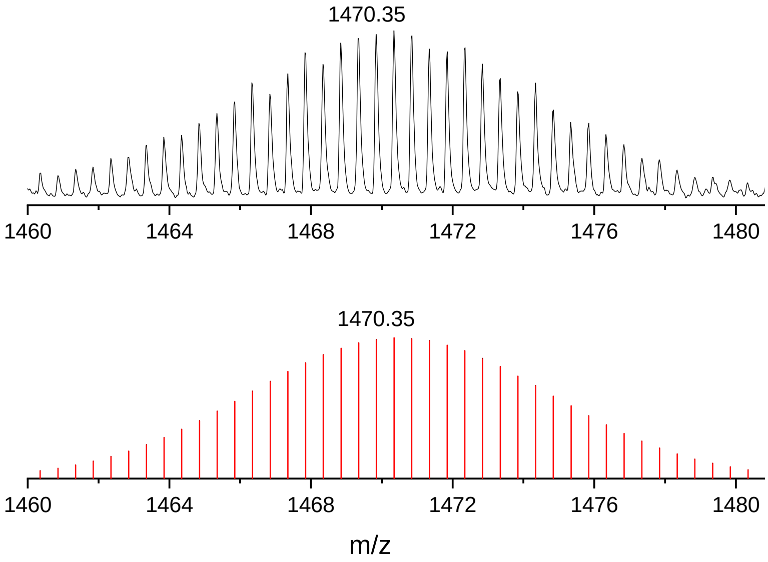

Supplementary Figure 9. Measured (top) and simulated (bottom) spectrum of the fragment {[K_2(2,2,2-crypt)][$Ge_{19}Pd_3(PPh_3)_3$]}$^{2-}$.

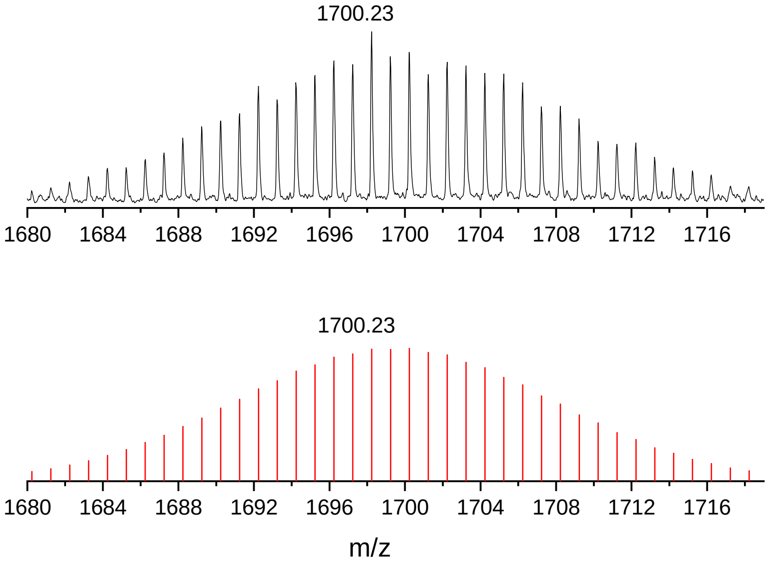

Supplementary Figure 10. Measured (top) and simulated (bottom) spectrum of the fragment {[$Ge_{19}Pd_3$]}$^{-}$.

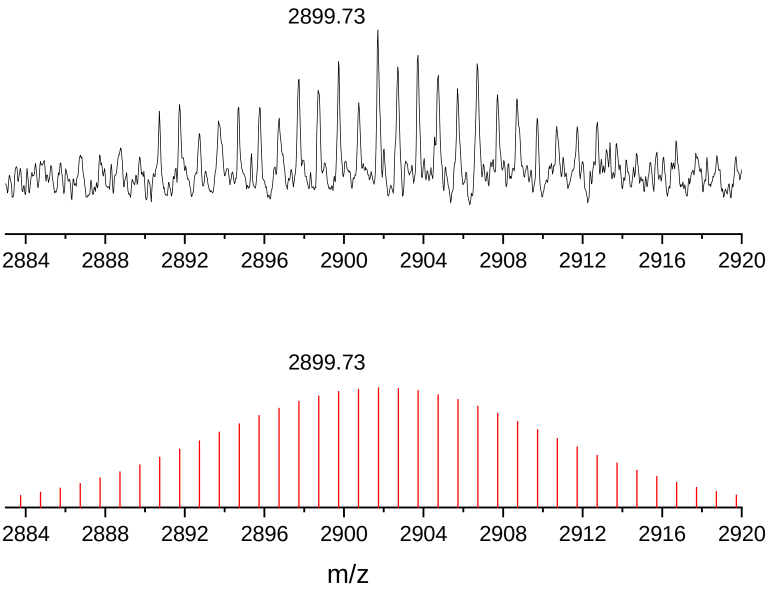

Supplementary Figure 11. Measured (top) and simulated (bottom) spectrum of the fragment {[K(2,2,2-crypt)][$Ge_{19}Pd_3(PPh_3)_3$]}$^-$.

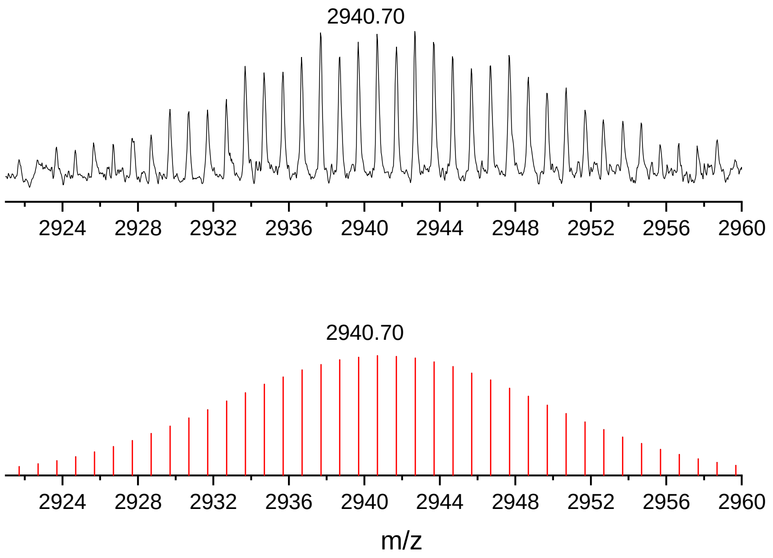

Supplementary Figure 12. Measured (top) and simulated (bottom) spectrum of the fragment {[K_2(2,2,2-crypt)][$Ge_{19}Pd_3(PPh_3)_3$]}$^-$.

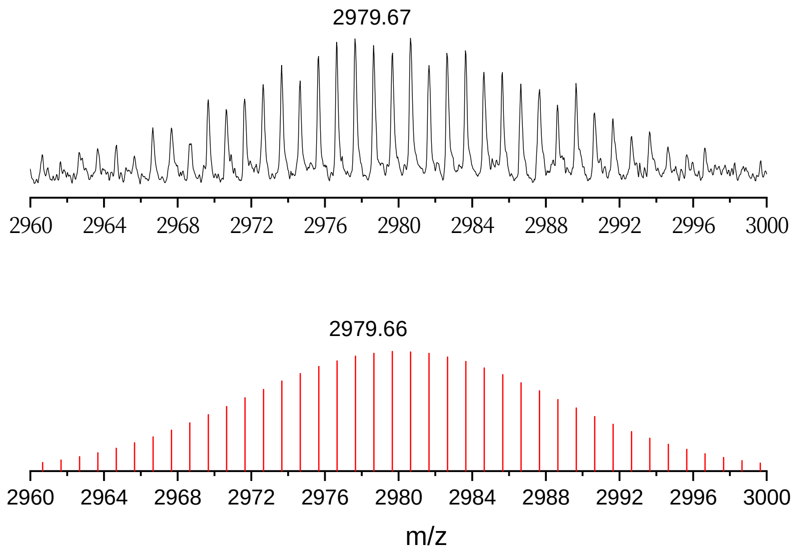

Supplementary Figure 13. Measured (top) and simulated (bottom) spectrum of the fragment $\{[K_3(2,2,2\text{-crypt})][Ge_{19}Pd_3(PPh_3)_3]\}^-$.

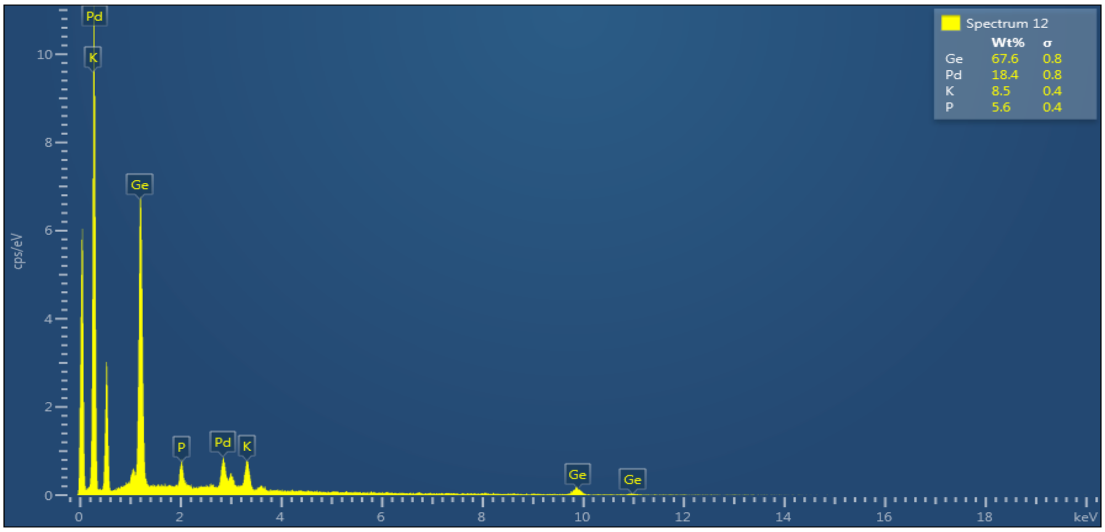

Supplementary Figure 14. EDX analysis of title compound (K, Ge, Pd, P).

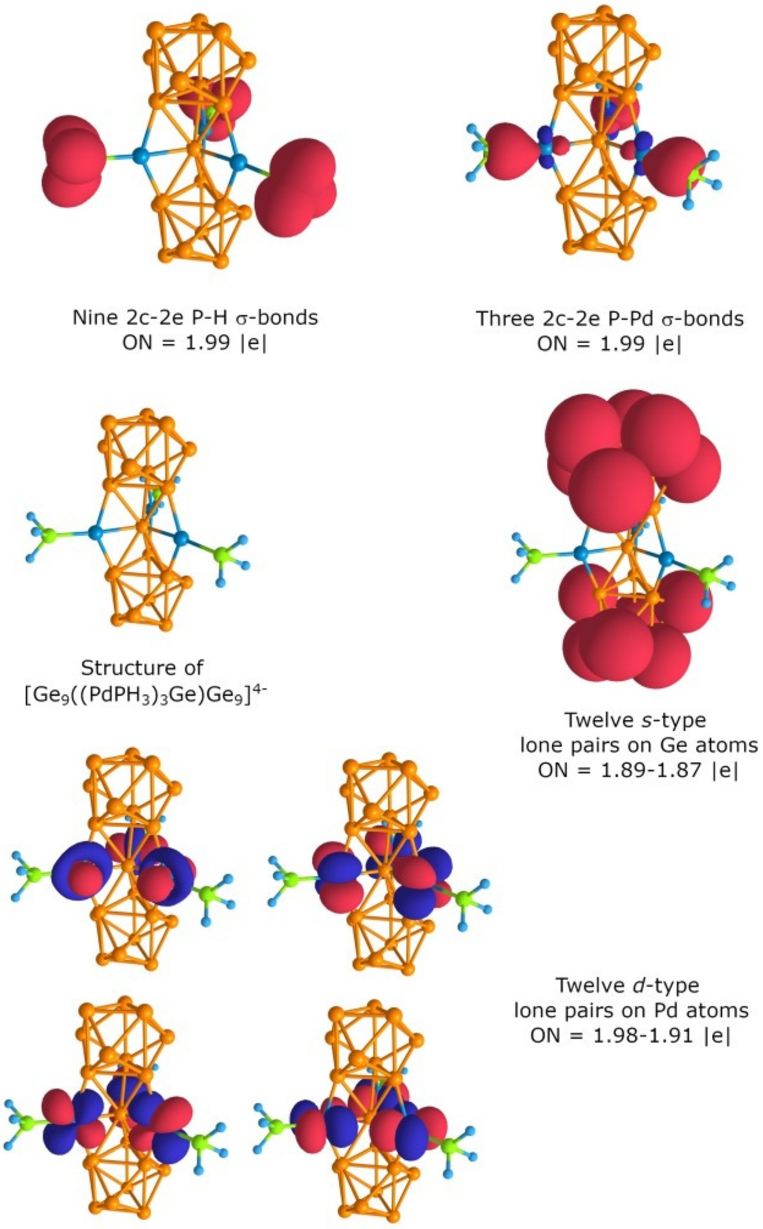

Supplementary Figure 15. Localized 2c-2e bonds and lone pairs of $\{(Ge_9)_2[\eta^6\text{-}Ge(PdPH_3)_3]\}^{4-}$.

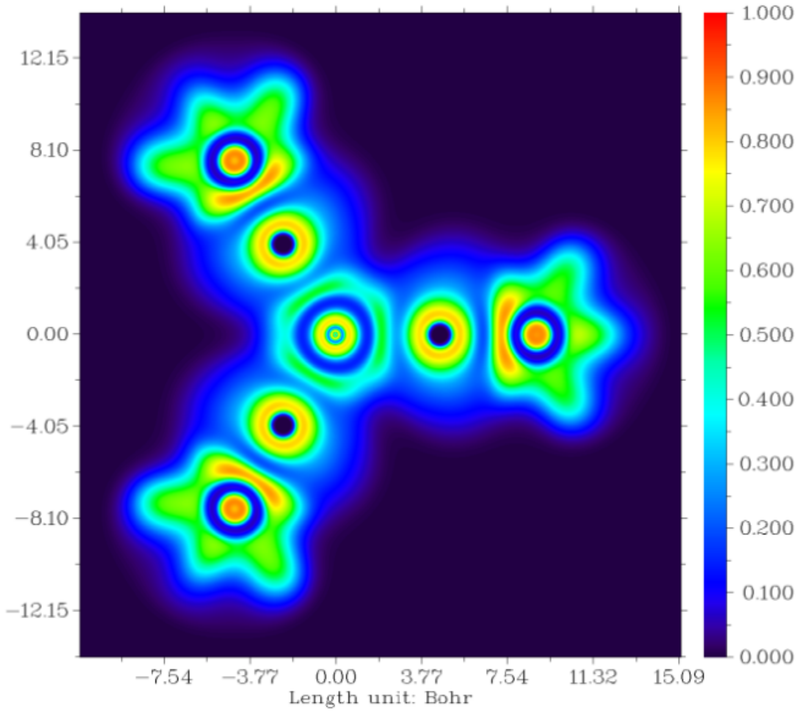

Supplementary Figure 16. ELF-plot of planar $GePd_3$ fragment. Different values of ELF are shown with different colors according to the color scale on the right side of the figure.

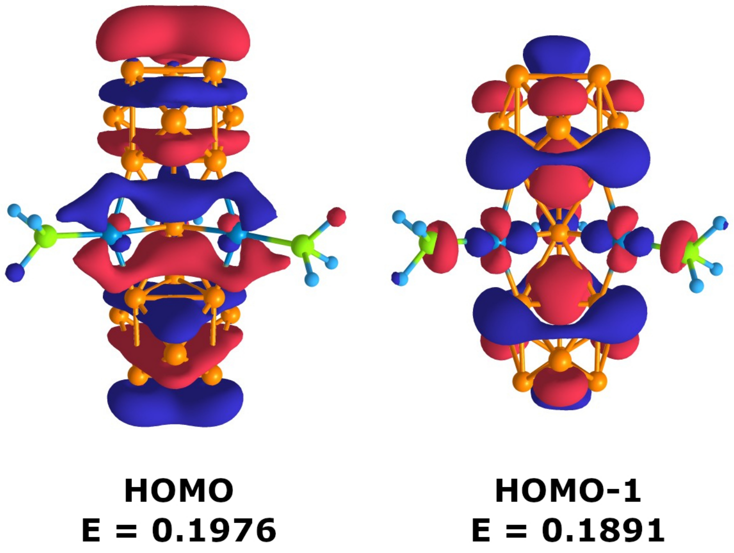

Supplementary Figure 17. Plots of HOMO and HOMO-1 molecular orbitals and their energies (Hartree) for $\{(Ge_9)_2[\eta^6\text{-}Ge(PdPH_3)_3]\}^{4-}$ cluster.

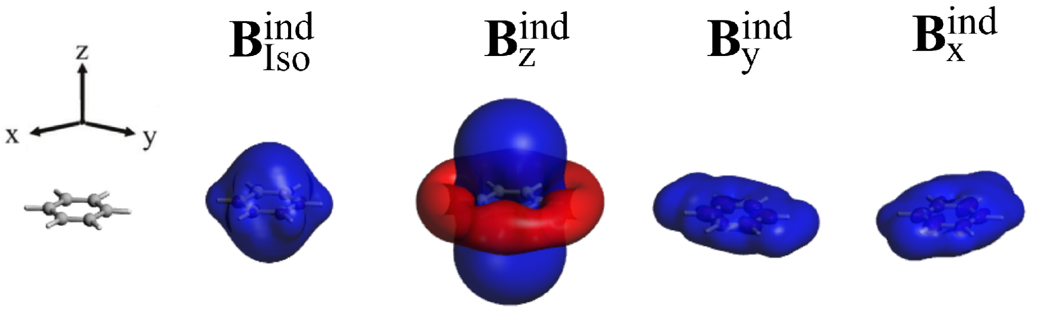

Supplementary Figure 18. The induced magnetic field for benzene.

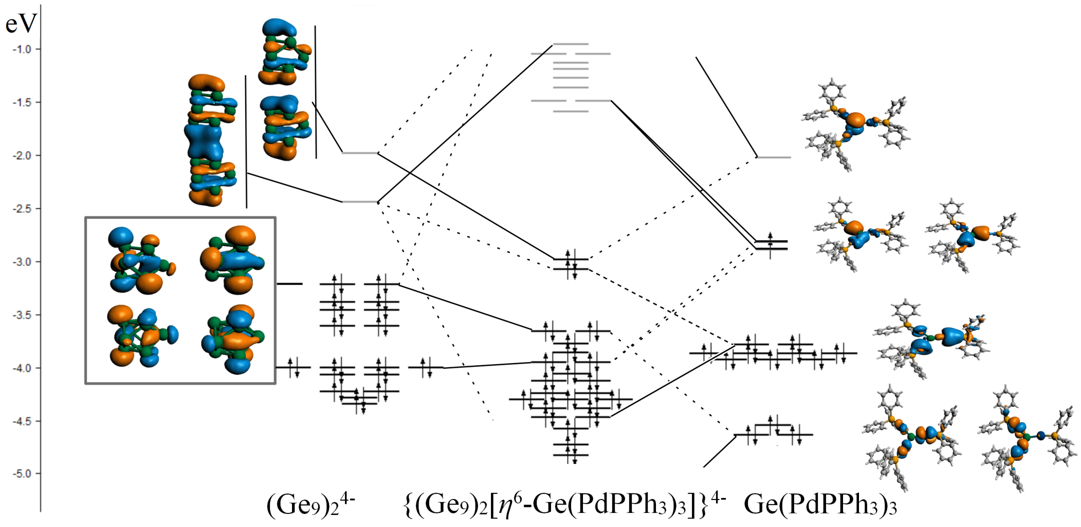

Supplementary Figure 19. Molecular orbital diagram denoting the $(Ge_9)_2^{4-}$ - $Ge(PdPPh_3)_3$ interaction. See Supplementary Figure 20 for HOMO-2 and HOMO-3 orbitals of the overall cluster.

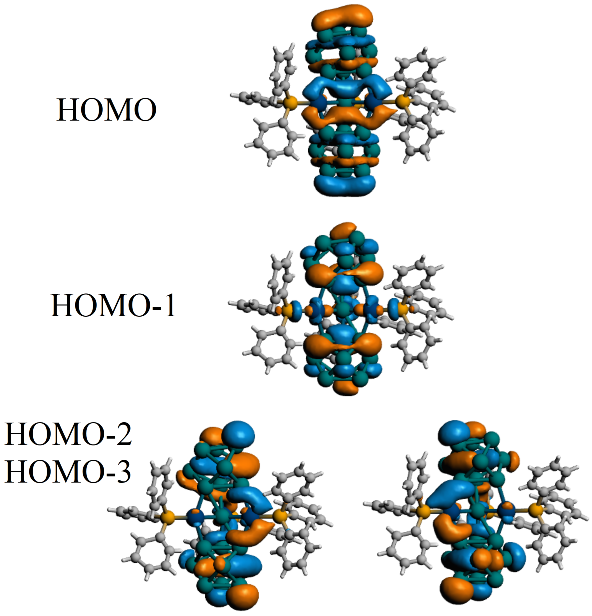

Supplementary Figure 20. Molecular orbitals for $\{(Ge_9)_2[\eta^6\text{-}Ge(PdPPh_3)_3]\}^{4-}$ cluster. Note resemblance to $\{(Ge_9)_2[\eta^6\text{-}Ge(PdPH_3)_3]\}^{4-}$ cluster (Supplementary Figure 17).

Supplementary Tables

Supplementary Table 1. Crystal data and structure refinement.

Identification code	$[K(2,2,2\text{-crypt})]_4\{(Ge_9)_2[\eta^6\text{-}Ge(PdPPh_3)_3]\}$
Empirical formula	$C_{126}H_{189}Ge_{19}K_4N_8O_{24}P_3Pd_3$
Formula weight	4148.46
Temperature/K	100.00(2)
Crystal system	monoclinic
Space group	$P2_1/n$
a/Å	32.6511(4)
b/Å	16.94251(18)
c/Å	63.3300(8)
α/°	90
β/°	97.7775(12)
γ/°	90
Volume/Å^3	34711.3(7)
Z	8

$P_{calc.}$/g cm^{-3}	1.585
μ/mm^{-1}	7.618
F(000)	16456.0
2Θ range for data collection/°	4.71 to 132.2
Reflections collected	171092
Data/restraints/parameters	60441/9473/3358
Goodness-of-fit on F^2	1.033
Final *R* indexes [I>=2σ (*I*)]	R_1 = 0.0957, wR_2 = 0.2334
Final *R* indexes [all data]	R_1 = 0.1133, wR_2 = 0.2431
Largest diff. peak/hole / e Å^{-3}	2.36/-1.66
CCDC	1997656

Supplementary Table 2. The calculated natural charges of the selected atoms.

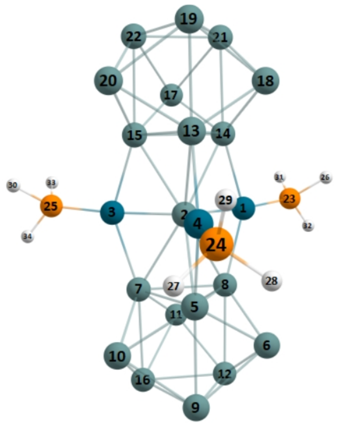

Atom	No	Charge
Pd	1	-0.39034
Ge	2	0.13383
Pd	3	-0.39034
Pd	4	-0.39075
Ge	5	-0.13035
Ge	6	-0.21708
Ge	7	-0.11487
Ge	8	-0.11487
Ge	9	-0.21029
Ge	10	-0.21708
Ge	11	-0.21608
Ge	12	-0.20795
Ge	13	-0.11177
Ge	14	-0.12743
Ge	15	-0.12743

Ge	16	-0.20795
Ge	17	-0.21804
Ge	18	-0.2169
Ge	19	-0.20663
Ge	20	-0.2169
Ge	21	-0.20934
Ge	22	-0.20934
P	23	0.21237
P	24	0.21223
P	25	0.21237
H	26	-0.02598
H	27	-0.02564
H	28	-0.02564
H	29	-0.05492
H	30	-0.02598
H	31	-0.02588
H	32	-0.05457
H	33	-0.02588
H	34	-0.05457

Supplementary Methods

ESI-MS Studies

Negative ion mode ESI-MS of a DMF solution of crystals of $[K(2,2,2\text{-crypt})]_4\{(Ge_9)_2[\eta^6\text{-}Ge(PdPPh_3)_3]\}$ was measured on an LTQ linear ion trap spectrometer by Agilent Technologies ESI-TOF-MS (6230). The spray voltage was 5.48 kV, the capillary temperature was kept at 300 °C and the capillary voltage was 30 V. The sample was prepared inside a glovebox and very rapidly transferred to the spectrometer in an airtight syringe by direct infusion with a Harvard syringe pump at 0.1 mL min^{-1}.

Energy Dispersive X-ray (EDX) Spectroscopic Analysis

EDX analysis on $[K(2,2,2\text{-crypt})]_4\{(Ge_9)_2[\eta^6\text{-}Ge(PdPPh_3)_3]\}$ (Supplementary Figure 14) was performed by a scanning electron microscope (FE-SEM, JEOL JSM-7800F, Japan). Data acquisition was performed with an acceleration voltage of 15 kV and an accumulation time of 60 s. The atomic ratio of K/Ge/Pd/P is 4.2:18.3:3.3:3.4.
