# Supplementary material for: A sandwich-type cluster containing Ge@Pd3 planar fragment flanked by aromatic nonagermanide caps
Source: Nat Commun. 2020 Oct 20;11:5286. doi: 10.1038/s41467-020-19079-z (PMC7575547; doi:10.1038/s41467-020-19079-z)
Supplement: Supplementary file 2 — Description of Additional Supplementary Files [file 41467_2020_19079_MOESM2_ESM.pdf]

Description of Additional Supplementary Files

Title: Supplementary Dataset 1

Description: Selected bond distances (angstroms) and angles (degrees) of cluster-1 and cluster-2.”
